# Parenting of Children with Autism Spectrum Disorder: A Grounded Theory Study

**DOI:** 10.3390/healthcare9070872

**Published:** 2021-07-12

**Authors:** Clara Roquette Viana, Sílvia Caldeira, Margarida Lourenço, Amélia Simões Figueiredo

**Affiliations:** 1 Centre for Interdisciplinary Research in Health, Institute of Health Sciences, Universidade Católica Portuguesa, 1649-023 Lisbon, Portugal; roquetteviana@ucp.pt; 2 Institute of Health Sciences, Nursing School, Universidade Católica Portuguesa, 1649-023 Lisbon, Portugal; margaridalourenco@ucp.pt; 3 Centre for Interdisciplinary Research in Health, Instituto de Ciências da Sáude, School of Nursing-Lisbon, Universidade Católica Portuguesa, 1649-023 Lisboa, Portugal; simoesfigueiredo@ucp.pt

**Keywords:** autism spectrum disorder, qualitative research, lived experiences, nursing research

## Abstract

Background: Parenting a child with an autism spectrum disorder (ASD) involves several processes and emotions during this transition. In addition to the family’s natural transition when a child is born, the family of a child with ASD has to deal with the particularities of the disability, its characteristics, and its evolution. Methods: This is a qualitative grounded theory study aiming to deepen the knowledge about the process of parenting children with ASD. Data were collected using interviews and observations of nine couples and one single mother. Results: Coding and analysis led to the main theme, which is as follows: parenting of children with ASD as representative of the parents’ transformation while caring for the child, also based on adaptation throughout this experience. Conclusions: Parenting is a dynamic process, grounded on the interaction of different contexts, such as family, education, health, and society, and on the co-construction of different times and episodes. These characteristics underline the complex and individual nature of parenting children with autism, which requires specific assessments and interventions by nurses when caring for these families, whether in a family nursing context, community nursing, and pediatric nursing or midwifery.

## 1. Introduction

Parenting is one of the relevant processes that occurs in the family life cycle and leads to a recognition by families of what has changed, in addition to the way in which relationships and circumstances are different [1,2]. Described as a complex transition process, parenting requires learning new topics, gaining new skills, changing behaviors, and, sometimes, changing self-perception and awareness in a social context [2,3,4]. When the birth of a child with differences occurs, the stages of family development cycle also change, based on adaptation processes. The place a child takes in the family is determined by parents’ expectations, but the birth of a child with differences challenges all the expectations of the parents and family, leading to a natural crisis period.

As parenting is a focus of attention in the nursing discipline and in nursing care, the study and development of effective interventions aiming to guide and support the skills of parents seem critical in person-centered care. Therefore, understanding the processes of parenting of different/disabled children seems critical to advancing this endeavor. Understanding the transitions, as lived by the patients or families, may guide nurses in providing adequate interventions and in developing knowledge on the topic. Nurses, doctors, other providers, advocates, administrators, and policy makers may need to understand parents’ feelings when they face the news of their child’s disability; parents’ feelings on having a different/disabled child. Examples of these feelings include parents’ primary concerns and wishes, parents’ expectations about external help, and parents’ strategies for parenting in the face of adversity, such as having a child with autism spectrum disorder (ASD).

ASD is considered one of the most serious disorders that can affect a child, in terms of both the affective and cognitive dimensions [5]. The developmental disturbance triad—communication, social interaction and understanding, and imagination—was first studied by Lorna Wing and is widely known as the “Lorna Wing triad”, and is a triad of impairments in autism [6]. Clinical diagnosis is based on the direct assessment of children’s behavior. The way in which autism interferes with development leads to living in an individual who is in their own world. These children have a very specific way of thinking and functioning that is characterized by difficulty in understanding and responding appropriately to the environment, selecting and processing information, and responding to other sensory stimuli. In this regard, very specific difficulties may be identified in these children, such as impaired social relationships, communication/language issues, and reduced interest in regular activities. As such, ASD is a development disorder that is considered extremely disabling and may bring stress and anxiety to parents This study aims to understand the process of parenting children with autism spectrum disorders, to understand the meanings and expectations experienced by parents during the process, to identify which critical events are highlighted by parents throughout this process, to identify the facilitating and inhibiting conditions or factors, and to understand the patterns of response by the parents in their parenting roles. The findings of this study will help nurses, whether in a family nursing context, community nursing, pediatric nursing, or midwifery, as well as other healthcare or social providers, to develop optimal care plans to target the specific needs of individuals with ASD and their families, which may require specific knowledge provided by a qualitative approach and descriptions by families that live under these circumstances. 

## 2. Materials and Methods

This qualitative study used a grounded theory method [7,8]. First, a characterization of the nature of the phenomenon under study was performed, distinguishing the object of study as a complex human reality, encompassing a system of relations with a dynamic structure and comprising phenomena with a procedural nature. As such, an interpretive paradigm, which comprises human subjectivity, was used.

### 2.1. Participants and Recruitment

Participants were enrolled based on a theoretical sampling process [7]. First, the study included an exploratory phase, aiming to access potential participants. This phase included exploring all Portuguese support associations for families of children with disabilities, based on website searches, conducted in November, 2015. A total of twenty-four associations were listed, and three supported access to eligible participants. These three associations were selected according to the following two selection criteria: the location—as data collection was planned to be done in Lisbon and Tejo Valley; and intervention—as the study aimed at interviewing parents with children up to six years old, which is the limit of early intervention, according to Portuguese healthcare guidance. These associations acted as intermediaries between the researchers and the families in the first stage. The inclusion criteria were shared with the associations and were as follows: parents of a child with ASD; members of associations in support of children with disabilities; parents willing to participate in the study in the first six years of their child’s life; children that do not present any other diagnosed morbidity, eliminating the potential that other factors may interfere with an understanding of the parenting process. A total of ten families were selected by these three associations, and provided consent to share their contact information with the main researcher. Then, all eligible participants were invited to participate, which comprised a theoretical sampling procedure, and all participants signed the consent form to participate. One family undergoing a divorce and only the mother participated, which was the parent with who the child lived. In the other families, the couple was considered the unit of analysis in data collection.

### 2.2. Data Collection Procedures

Data collection was based on observations and in-depth interviews that were conducted at families’ homes, which was the daily environment where experiences take place [9]. Making contact with the family in a home-environment context also facilitates obtaining a deeper understanding of the physical conditions where they live and provides an opportunity to cross paths with family and friends, giving access to the family’s social experiences, which provided a better understanding of family dynamics and factors that may affect parenting.

Each family interview was given the name of a star, as each of the stars in the universe are unique, which was also the case for each family, as evidenced by Acosta and Vitale [10], who referred to them as a constellation of people. In addition to interviews, observations of parent/child/family interactions, environment, and context were also conducted. The interview comprised two primary components: the first, was to get demographic data and to bring together the interviewer and participants in their homes and the second was the moment when all questions about the phenomenon under study were asked. These were open questions, aiming determine experiences, perceptions, and attitudes towards having a child with ASD, namely, how parenting is lived, what it is like being the parent of a disabled child, how (and who) was involved in nurturing the child, and what the journey of becoming parents was like when a child is different to what was expected. The interviews lasted from 55 min to 120 min, with an average of 90 min. All interviews were taped and manually transcribed verbatim. 

Observations were made based on a guide for interactions with a mother/father/child, related to parenting. The observations were based on Spradley [11] and the Dynamic Model of Assessment and Family Intervention [12], with specific reference to the Functional and Development Assessment, to help understand a family as a complex and multidimensional system. 

This study was approved by the Institutional Ethics Committee (ARSLVT process—9787/CES//2015), and all parties signed an agreement to collaborate. Informed consent was obtained from all participants. 

### 2.3. Data Treatment

Constant comparative analysis is a critical characteristic that guides data collection in grounded theory research, and includes comparing and classifying incidents, as well as a theoretical comparison in order to stimulate thinking regarding the properties and dimensions and to guide theoretical sampling, aiming for a better understanding of all findings [7]. Interviews were transcribed to and double checked by two authors prior to analysis, interpretation, and theorization. 

## 3. Results

### 3.1. Participants’ Characterization

The sample was composed of ten women (mothers) and nine men (fathers). Of the ten families, eight were nuclear; six parents were married (the average age of mothers was 34.8 years, and of fathers was 36.5 years); fifty percent (50%) of mothers had a higher education level and 60% of parents had a secondary education level. In relation to their professional activities, five mothers were exclusively dedicated to the family, while the other five engaged in regular professional activities; all parents had a job. As for the number of children, the mode was two children. The household of these families resulted in an average of 4.1 people/family. Eight families planned the pregnancy and all families had prenatal care. Four mothers had complications during pregnancy and two couples submitted to assisted reproduction technology. Six women had a cesarean section. All were term children with Apgar scores between 8 and 10; seven were male and three were female. Eight of the mothers were multiparous. The children in the study were aged between two and a half years and six years. The age at which they were diagnosed with ASD ranged from two years to five years of age. 

### 3.2. Qualitative Analysis

**Figure 1:**
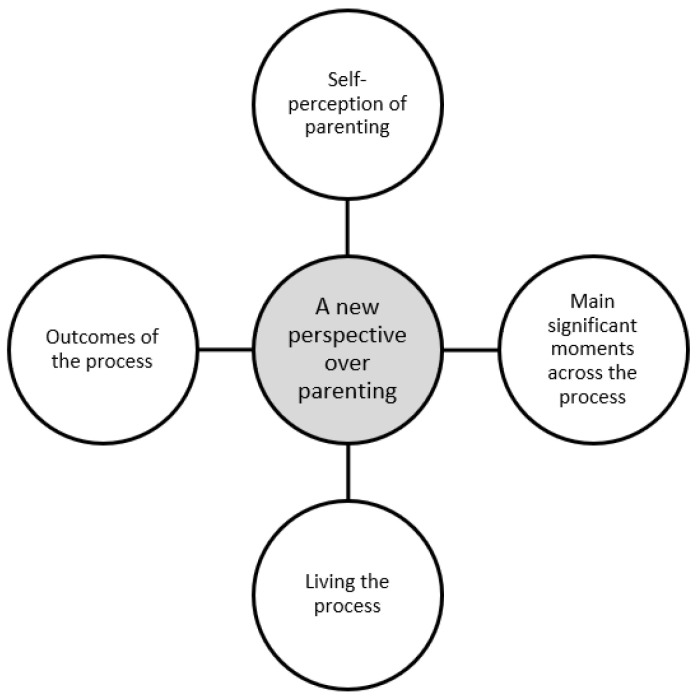
Original main categories found in the study that compose the main theory of “a new perspective over parenting”.

The analyses of the findings from the interviews with the parents highlighted a new perspective regarding parenting, inherent to the experience of being a father and mother of a child with ASD, and was organized into four categories: “self-perception of parenting”, “main significant moments across the process”, “living the process” and “outcomes of the process”, which characterize the nature and the parental process in this particular experience (Figure 1).

#### 3.2.1. Self-Perception of Parenting

An analysis of the interviews with the parents highlighted essential issues regarding self-perception of parenting in the context of experiences with these children, such as the meanings, expectations, and socio-economic status (Table 1). 

The meaning given by parents regarding the concept of disability is commonly described as to “start living in a different world”, a world where life is lived in a different way by letting go and changing priorities. Perceiving disability as not inherent to the child, but rather as a complex set of conditions, many of which are environmental, moves the perception of disability to the concept of difference. Another word that was often used by parents in the interviews was “special”, in the context of emancipating the children and removing the influence of the word “disabled”.

Similarly, when approached about ASD, word singularity was highlighted, as each child was described as being unique. Even using different terms, all parents referred to the individuality or uniqueness of their own child, despite the ASD. The characteristics of behavior varied widely from child to child, and in the same child over time. Some symptoms may be more revealing and intense at one age but may change in nature and severity at another age, leading to very different clinical profiles.

All participants referred to ASD as a complex and mysterious condition. The inaccuracy of stereotypes of autism as well as its nature are factors that contribute to this mystery. Confirming the diagnosis of ASD confronts a family with a chronic disorder with an unclear etiology. This is more specific information, but still does not cover all that parents may need and want to know. 

The major concerns of the parents related to an awareness of their own finitude, the child’s needs for care, and perceiving the scarcity of institutions that can support/care for these children as they age. The findings disclosed that expectations of the future comprised the prognosis, the possibility of dependence, and social discrimination. When talking about perceptions of the autonomy of the children in the future, as independent adults, parents revealed that are aware of the difficulties in the development of these children and in achieving full autonomy, but they hope that the future may bring alternatives in terms of quality of life. The expectations of most parents revealed a commitment to their children to reach a higher level of autonomy and development. These expectations were nurtured by a sense of hope for the future. Parents tried to identify objects of hope and set clear goals, examine and assess reality in relation to hope, which include assessing all factors (internal and external, potential and real) that may comprise hope, have a perception of realistic desires for the future—which are possible but uncertain. These perspectives influence the sense of physical, emotional and spiritual well-being. 

It is widely accepted that ASD has an important impact on the lives of parents and the family in an emotional and dynamic context, but also from an economic dimension. This aspect of additional costs was mentioned by families when referring to the diversity of therapies and support that the children needed, which is also important in the context of parenting. Economic difficulties are stressful and constitute a risk factors that families need to deal with, which affect the patterns of parent–child interactions. Additionally, leaving job responsibilities and roles to be able to take care of the child full time, losing moments of leisure, and everything that includes time and restrictions in the family’s life, impact economic balance that is also critical in parenting, particularly under these conditions. Socio-economic status generally refers to income, level of adequacy and education of family members, and the social level resulting from salary. Families with a high socioeconomic status have a greater amount of resources to cope with a disability when compared to a family at a lower income level. Families with a higher socioeconomic status reveled higher expectations and so, shared a greater impact of having a child with ASD. 

The interviews disclosed that, regardless the socio-economic profile, family values are also foundational in maintaining structure and may deeply interfere with the way the child with ASD affects the entire family. 

#### 3.2.2. Main Significant Moments across the Process

The transition to parenting is a change at different levels, requiring an adaptation process to carry out a set of specific development tasks, leading to a redefining of identity and roles. The following categories emerged in this regard: birth, confrontation with a different form of behavior, facing reality (the diagnosis), awareness of health, and awareness of education.

In the birth category, there are two subcategories, pregnancy/childbirth and breastfeeding, events that constitute significant changes in the life of a woman/couple, leading to a change in roles. Although pregnancy and childbirth, as well as breastfeeding, are unique and positive experiences, parents recognize their new status and demands in the context of the different behavioral, emotional, and cognitive responses. 

They also admit that nurse interventions are very important and can contribute to the experience of the process, depending on the approach.

In the category of confrontation with different behaviors, four subcategories arise: failures in communication/interaction, stereotypes that describe changes in the child’s behavior and that are evident in development and social interaction, parents’ concerns, and devaluation opinions regarding the forms of behavior. 

The presence of all doubts and uncertainties resulted in an added stress factor for families, increasing the sense of vulnerability in the adjustment to the unknown. However, there is great perseverance on the part of parents in understanding the needs of these children in order to find meaning for their interventions. Their feelings, the intuition that something was not right, led them to seek answers for a better understanding of that phenomenon.

The category of meeting with reality arises from the confrontation with the diagnosis. In this category, subcategories of shock and relief, which stand out from a succession of events that are part of the grieving experience, have been found in the context of the imagined child. This question was sensitive to the parents; however, the impact of the news was also perceived as a relief in the face of so many uncertainties and, simultaneously, as a way of facing the condition.

Mothers seemed to be predisposed to have greater difficulty in individual adjustment and fathers showed greater concern with the stigma of the condition, along with the future and the subsistence of the family. 

In the category of awareness of health, three subcategories were highlighted: a lack of response from professionals, communication failures between services, and the scarcity of resources. The experiences revealed a lack of response on the part of the professionals, which delayed diagnosis and made it difficult for to understand the behavior of these children. The participants’ perceived a lack in communication between health services, the organizations, and dynamics of the therapeutic process.

Parents recognize the feeling that they are unable to, due to various demands, and lack sufficient knowledge to establish the priorities of a therapeutic plan and require guidance during the process. In addition, in their opinions, the available resources were scarce and not adequate to the demands of the children.

The category of awareness of education highlights the subcategories: little involvement of teachers, scarcity of educational resources, and recognition of the vulnerability of children in schools. Parents considered that the human resources were not fair in comparison with needs, namely in the transition of the children from pre-school to primary education. Parents reported that there was no continuum in the educational process, even though professionals are unaware of the functional content of both services. At the same time, they were faced with poor preparation by the schools in relation to this issue (ASD) and with little knowledge and involvement by teachers to meet inclusive practices.

Finally, they still face concerns about the acceptance of their children in school, which was described as a stressful factor. 

#### 3.2.3. Living the Process

Living the process implies the series of changes in a family’s life that arise from the awareness of a child being different, accompanied by the characteristics of the process.

Changes in life take place throughout the process with the achievement of a new perspective on disability. Adaptation to a new way of living, changes to the couple’s relationship, and changes to social and professional lives produce a sequence of transformative events that emerge, support, and mediate the transition. 

These changes meet the acceptance of a child who is different and goes through the grief experience of an idealized child. Awareness influences an increase in knowledge and involvement, reflecting parents’ efforts to better understand their new role and rebalance themselves in the context of the new situation. The individual feeling of being connected and interacting with their situation is one of the indicators that a transition is taking place, translating into the development of growing confidence in adapting to change and in acquiring new skills and ways of life that contribute, simultaneously, to a new sense of identity.

The recognition of a child’s behavior also led to the adoption of strategies as resources and internal mechanisms, with the purpose of guaranteeing or preserving the quality of life of the child and the family. These strategies promote progressive family adaptation, leading to the development of parenting skills and a reorganization of the family.

Parents emphasized that living with their children made them reevaluate their values and priorities in an attempt to assign meaning to them: strengthening spiritual or religious beliefs; increasing the ability to focus on the present and the ability to appreciate simple and regular things in daily life.

It was evident in the interviews that there was a search for knowledge through various means of information, which contributed to greater involvement in the care of the child (an emphasis was on easy access through the Internet). Another source of information was healthcare professionals.

One of the problems that parents experienced through social interactions was stigma. Restrictions on social contacts contributed to creating greater tension and social isolation. On the one hand, some families moved away from a social life, and, on the other, some families continued cultivating social relationships. Most families included the child with ASD in their leisure activities, although there was a unanimous opinion on the difficulty of integrating children into these experiences, justified by the child’s behavioral problems and the reactions of society in a public context. ASD remains a developmental disability that is highly stigmatizing and has an impact on family dynamics.

The support network of an extended family is a major external resource. The mutual assistance verified in these families was more occasional than systematic, functioning basically in a network of restricted kinship and female mutual assistance.

Another referenced resource was circles of friendship. Most families said that they did not have support from friends, because the families were intimidated by the experience of the situation, and they moved away from their circles of friends.

In seeking institutional support, parents turned to national associations. This type of institution was considered to be of great importance in monitoring children and families and in raising awareness in society, with regards to citizens’ rights and opportunities.

This experience is lived differently by each family. The path that each takes and the time it takes to do so are unique. Some couples got closer and found the crisis to be a situation of growth and maturation. The mother was the one who monopolized the responsibilities for the child and fathers usually spent more time away from home at work. However, both were described as dedicating time to childcare, often neglecting marital relationships and social activities. It was the two-parent families that felt most prepared to educate a child with ASD, supporting each other in parenting.

Disability is characterized as difficult, time-consuming, and challenging, even though everyone recognizes it as unfinished—a continuum in a process of self-transformation.

#### 3.2.4. Outcomes of the Process

The changes resulting from experiencing the transition process reflected the way in which parents were able to overcome difficulties and challenges and to find appropriate strategies to adapt to the new role. 

Aspects, such as self-knowledge and lived experience, which emphasize the valorization of coexistence and sharing, supported the feeling of overcoming challenges, the sense of stability and tranquility, personal growth, and the full performance of the parental role. The couples gradually became the father and mother to a child with differences, following their own trajectories and respecting each other’s inherent time. However, the conception of a common project was always present in interviews, revealing a confluence of ideas and concepts that determined the growth in their parenting.

The way of thinking and acting showed a whole process of transition, of deep integration of change, and complex adaptive activity. Parents recognized being different people, with ruptures and identity reconstruction, supported by the experience of their new roles and responsibilities.

This reconstruction is associated with successful adaptation to parenting, occurring simultaneously with the development of self-confidence and autonomy in performing the parental role. The transition, as experienced by these parents, can be seen through the redefinition of their roles, a mastery of new skills, new relationships, and the management of emotions. All these come to a sense of being relieved by increasing feelings of satisfaction, confidence, autonomy, rationality, and control.

The interviews allowed the sharing of stories and a symbolic universe, which are organized, confer uniqueness, are complex and promote the experience of parenting. Life projects are associated with the culture and fundamental values of the parental identity that are reaffirmed, rebuilt, and relearned in the presence of a succession of events and the specificity of the experiences that accompany the reality of each of these families.

**Figure 2:**
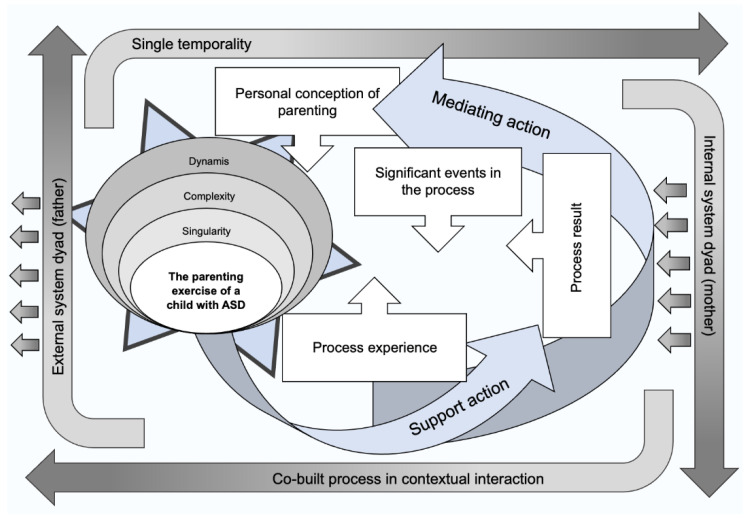
Synthesis of the theory entitled “a new perspective over parenting” that resulted from this grounded theory, that focused on parenting children with autism as a dynamic journey, grounded on the interaction of different contexts, such as family, education, health, society, and on the co-construction of different times and episodes.

Parents live a process of remaking identity, which is associated with a redefinition of priorities and a greater sense of responsibility. This whole process led to greater confidence in parental performance and to a greater awareness of changes in personal development. All these findings were merged and analyzed, and are the foundation of a new perspective on parenting, as represented in Figure 2.

## 4. Discussion

A new perspective of parenting is the central theme that emerged from the study and, through the connection between the four identified categories, generated the central idea of the phenomenon. This resulted from interpretation of the findings, obtained from the testimony and experiences of the parents who participated in this study.

When validating the theoretical explanation, an interactive construction process began with the birth of the child that emphasized the temporality (as a succession of events) of the parental condition, since it constitutes a reference in the parents’ lives. Concomitantly, the specificity that surrounds this experience converts the experience into a unique, dynamic, complex process equipped with great challenges, [5,13,14,15,16] leading parents through the process to a new perception of the concept, translating it into a new way of thinking, feeling and acting.

The integration of the categories in the circle of the central category, and in the relationships between them, leads to an understanding of the chronology of events. The exercise of parenting develops over time through living with the child, where there is a succession of integrating events and where the process is characterized by continuous learning, changing and developing relationships, and by a profound renewal of the self [4,10,14,15,17]. This process of parenting proves to be a co-constructed process, inherent to the personal experiences of each parent and is involved in a very specific context that is the world of the spectrum of a child with ASD.

The beginning of the parenting process comes with the birth of the child and with the awareness of the countless changes that occur in their lives, inherent to their new situation [5,13]. This scenario generates ruptures in systems of meanings and reveals weaknesses in view of the need to redefine a new reality in life, a situation that intensifies when faced with the situation of a disability, such as being a parent of an child with ASD. The diagnosis of a family member’s disability has a very significant impact on the whole family [15,18]. The family dynamic undergoes profound changes due to special situations like this, from financial conditions, to issues related to the physical, psychological, and social quality of direct caregivers or parents [5]. The diverse experiences that parents face emerge as something new in their experiences, constituting a challenge to be overcome. Feelings also develop as personal experiences are generated by each parent and by both as a couple, integrated in the parenting experience [13,14].

In this theoretical construction, the strategies used, and the parental responses, to deal with parenting are the main pillars that both support and sometimes mediate the significant parental experience. They are interconnected human actions, which result from a dynamism presented by interactions and strategies, with the purpose of empowering parents in achieving their parental roles in the contexts that they experience [13,16,19,20].

The parents’ strategies to deal with parenting and the specificity of ASD, as well as self-restructuring, have various dimensions: cognitive, related to the learning of new skills, the redefinition of the parental role and decision making; relational, where family support and friends stand out; social, where emphasis is placed on the child and family support associations, health services, schools, and society; other components that were also highlighted by the parents refer to the operational field, where involvement in care, reorganization of habits, sharing of tasks and the reconciliation of roles stand out. All these elements contribute in order to favor the transformation of the concepts of life, beliefs, expectations, relationships, and day-to-day living [5,14,16,19,20,21].

The various conditions that interfere in the experience of the process, identified as intervening conditions in this case, unite parents in a more comprehensive structural context of their experience, manifested in a set of meanings, values and beliefs shared by society. It is through the conception of this culture that parents interact, act, and attribute meaning to the experience of the parenting process, mirroring the response pattern through fluid integration and mastery of competencies, in this developmental continuum [2].

Despite having developed a theory limited to a small reality, it can contribute to the development of a broader theory and be a starting point for other studies that complement and add knowledge to this area that is a vast field yet to be unveiled. Emphasis is on the importance of research focused on care and enables the development of knowledge about the world of human experiences. Giving visibility to these experiences and integrating them in healthcare is a way of moving towards the humanization of care and participating in the growth of nursing as a science of care, regardless the clinical context that it is related to.

## 5. Conclusions

Parenting requires different behavioral, emotional, and cognitive responses from parents, which implies a specific adaptation and reorganization of life, which can sometimes lead to situations of decompensation and the acquisition of vulnerabilities, especially in the face of an adversity, such as having a child with ASD. The main theme of this study is a new perspective of parenting. Findings lead to the need for efficient and articulated multidisciplinary teams in providing support to these families in developing parental roles. Parenting a child with ASD is a dynamic process, fueled by the interaction within the various contexts, family, health, education, and society, based on a co-construction that is substantiated by successive temporalities (as a succession events), that covers the phenomena of complexity and uniqueness. Specifically, in what concerns nursing, these families may require specific assessment and intervention, whether a in a family nursing context, community nursing, pediatric nursing or midwifery. As such, policy planners should be aware that these parents need to embrace health, education, and social partners, such as associations, to work together on a global supportive answer to help parents in overcoming this situation and to help children in maintaining global development.

More research that includes children and parents, such as participatory action research, may be helpful to better raise children with ASD, supporting parents and getting more evidence on this specific population, health condition based on an evidence-based approach.

## Figures and Tables

**Table 1:** Findings: categories, sub-categories, codes and quotations *.

Categories	Sub-Categories	Codes	Quotations
Self-perception of parenting	Meanings	Deficiency To be different To be special	“*She is a special child, that is, more than having a disability she is different […] Takes more time, learns differently, at your own pace* (M)” (I7—3).
		ASD Singularity	” *In these children there are characteristics that are really common to all, but most of them are different and, even those that are present at the moment can change at times of life […] this is a real mystery* (M)” (I8—2).
	Expectations	Apprehension of the future	*“We know that he will not be able to live alone, he will always have to have support […] while we’re all right, the problem is when we’re not here anymore* (M)” (I6—4).
	Socioeconomic status	Economic difficulties	“*The values of therapies in the private sector are very high and those co-funded by the state are insufficient […] which makes everything more difficult* (M)” (I1—2).
Main significant moments across the process	Birth	Pregnancy/childbirth	“*During pregnancy we live in expectation, but when they are born it seems that they exceed that expectation […] it’s a wonderful emotion* (M)” (I3—1).
		Breast-feeding	“He really didn’t want to suck, it was a struggle from the beginning […] I came home and had to talk to SOS Breastfeeding ** because it was a mess, I didn’t know what to do (M)” (I5—2).
	Facing a different behavior	Communication/interaction failures	*“My son did not speak, although he made some sounds* (M)” (I5—3).*“I came home from work and she stayed the same, our presence was indifferent to her […] she even changed divisions when we were present* (F)” (I7—2).
		Stereotypes	*“He started to rotate objects, to stack them, to put them in a row, he didn’t play pretend* (M)” (I8—2).
		Parents’ concern	*“I was very worried because I realized that there was something […] I didn’t know what, but I felt, I knew he was not well* (M)” (I1—3).
		Devaluation of opinions	*“We talked about our suspicions, including thinking about autism, but they did not appreciate us […] we feel very alone* (M)” (I7—2).
	Meeting with reality (diagnosis)	Shock	*“It’s a bucket of cold water ”and we didn’t want to believe it […] we cried, […] we seem to have paralysed* (M) ” (I8—4).
		Relief	*“For us it was a rest because our greatest anguish was not knowing what was happening […] it was the confirmation of our suspicions and from then on we already knew the way to go […] it was the beginning of the mourning of that perfect son who we imagine having* (F)” (I6—5).
	Awareness in the face of health	Little response from professionals	*“It is all very bureaucratic […] not at all facilitating with regard to support, information, what is there and how to access it (F) […] I felt the lack of someone (doctor or nurse at the health center) to guide me […] The State should activate the support and they should direct us and we should not be the ones to have to look for them […] they should organize themselves among themselves (M)”* (I7—2).
		Communication failures between services	“*There is no coordination between the health services (doctor and family nurse with the hospitals and support services), there is no link in the monitoring […] this does not mean that the professionals are bad, the system does not work* (M)” (I10—1).
		Scarce resources	*“Consultations at the hospital to monitor [Polar Star] are annual, imagine only once a year, how is it possible? (M)” (I1*—*2).*
	Awareness in the face of education	Little professional involvement	*“Within the team itself there is no communication and teaching strategies […] there are educational guidelines in theory, but in practice it is a matter of luck […] the school does not try and invest in these children (M) […] The service does not is being done and resources are being spent* (F)” (I6—3).
		Child vulnerability recognition	*“We heard about abuse, mistreatment […] many times [EstrelaPolar] comes home with bruises, which is normal for any child, but I’m always afraid because he doesn’t speak, he can’t tell what passed and I only have one adult version […] it’s distressing (M)”* (I1—4).
		Scarce resources	“*There are few special education teachers and therapists in schools to meet the needs of these children* (F)” (I2—3).
Living the process	Life changes	A new look at disability Acceptance of a different child	*“The essential thing was to understand what was happening with the [Swan] […] was to go through that phase of denial and progressively accept and integrate this aspect in my life and family life […] it was being able to make options, stop, think and decide what you wanted to do with your life* (M) ” (I7—1).
		Adapting to a new way of life Learning Involvement in care Changes in habits/ Change in routines Prevention of embarrassing situations	“*Our daughter’s situation made us change a lot […] it brought us a new way of looking at life, lighter, with other values* (F) ” (I7—2).“*I changed the chip and I was able to combine the aspect of caring with the pedagogical aspect […] I gathered all the material I had, learned how to work with these children and advanced […] I worked a lot with [Swan], I was a mother, teacher, nurse, but it was worth it and it’s still worth it (M)* ” (I7—1).*“A lot has changed […] our routines have changed, so many things we would like to do (M) […] today we are a watch and Swiss (laughs) […] we have to adapt to it (F) […] there are many things that are delayed (M)”* (I4—2).
		Changes in the couple’s relationship Less availability in the relationship Conflicts Union/love	*“The fact that we support each other and are together is very important in the relationship* (F)” (I7—3).
		Changes in social life Isolation Concern about maintaining social life	“*[Polar Star] has some manifestations of contentment that are recognized as antisocial […] the parents of the other children are looking at me as if I were a “denatured” mother […] it is very complicated to manage these situations […] everyone looks at us (embarrassment)* (M)” (I1—2).
		Changes in professional life Option to suspend professional activity	“*It was also the moment to decide to stop working because it was very difficult to reconcile work with the answers I had to give at home […] at that moment the priority was [Swan] and she needed me full time (M)”* (I7—3).
	Process characteristics	Internal resources/mechanisms Grief experience Spirituality	“*Nowadays I see life in a different way, I see that there is a lot to be lived [...] I rely on something that is fundamental to me, which is Love [...] the important thing is to have the strength to be able to provide happiness to my children, and we feel happy parents too* (M)” (I4—2).“*Strength comes in everyday life […] is a new way of being and seeing the world […] (M)”* (I6—2).“*The strength comes from inside (M)*” (I8—1).
		External resources/mechanisms Family/Friends Association Health and educational therapies/services Society	“*I’m always saying that I never needed my mother as much as I do now, she always puts her hand on me when I need to* (M)” (I3—1)“*We stopped going out frequently, but we keep in touch with a couple who are friends for life […] We are more intimate and comfortable* (M)” (I8—2)“*Thank God we have had the Association with people who have been extremely important throughout this journey and have guided us in a spectacular way* (M)” (I3—2).
Outcomes of the process	Self knowledge	Self/personal competence	*“We have learned so much from this whole situation (M) […] from what we have learned we have adopted our way of dealing with him and we think that this is the best approach to [Belatrix], which works best with him […] We were able to assess his learning and the relationship he has with us so we will continue along this line, although we are always open to experimenting with other approaches (F)”* (I3—3).
	The experience	Coexistence	*“The books talk about autism, but they do not teach how to deal with these children […] only through daily coexistence […] from the experience […] do we really understand and learn […] you need to be there and there is no one better that parents (M)”* (I1—1).
		Share	*”The existence of support groups is very important […] parents have so many things in common and do not always have the opportunity to relate to each other and exchange precious ideas […] not even our families perceive us as well as others parents living similar experiences (F)”* (I2—1).
	Mastery of new skills	Satisfaction and confidence in the performance of the parental role	*”This effort on our part and the investment made has had rewards and today I look back and realize that we took the right option and the important thing that was for the development of [Swan] (M) […] all the moments of the journey were important, they were an apprenticeship and it is for them that we continue and struggle continuously […] we are confident and optimistic (F)”* (I7—1).
		Efficiency	*”Knowing how to anticipate some behaviors, knowing what we should do to make them more balanced, more calm is constant learning (M)”* (I10—1).
	Redefining parental role	Personal growth	*“I think I becme more human, calm, having more resilience despite all concerns […] I can manage better all situations I feel stonger (M)”* (I1—1).
		Improvement in achieving parental role	*”Nowadays I see life differently, I see that there is much more to be lived [...] it is very difficult to have a child with special needs, but nowadays I no longer dramatize, I see life in a simpler way […] the important thing is to have the strength to be able to give happiness to my children, and to feel happy parents (M)”* (I4—2).

* A Supplementary Table S1 is available with these same results in Portuguese. ** Helpline to support mothers in breastfeeding provided by nurses or midwives. Legend: M—Mother; F—Father. The code after quotations refers to the interviews and codes of the analysis.

## Supplementary Materials

The following are available online at https://www.mdpi.com/article/10.3390/healthcare9070872/s1, Table S1. Findings: Categories, sub-categories, codes and quotations in Portuguese.
