# Supplementary material for: Parenting of Children with Autism Spectrum Disorder: A Grounded Theory Study
Source: Healthcare (Basel). 2021 Jul 12;9(7):872. doi: 10.3390/healthcare9070872 (PMC8306821; doi:10.3390/healthcare9070872)
Supplement: Supplementary file 1 [file healthcare-09-00872-s001.zip › healthcare-1209500-SI.pdf]

Supplementary Table

Table S1. Findings: Categories, sub-categories, codes and quotations in Portuguese.

Categories	Sub-categories	Codes	Quotations
Conceção Pessoal da Parentalidade	Significados	Deficiência<br>• Ser Diferente<br>• Ser Especial	"Ela é uma criança especial, ou seja mais do que ter uma deficiência ela é diferente […] Leva mais tempo, aprende de forma diferente, num ritmo próprio (M) " (E7 – 3).
		PEA<br>• Singularidade	"Nestas crianças há características que são realmente comuns a todas, mas a sua maioria são diferentes e, mesmo as que têm presentes no momento podem-se alterar em alturas da vida […] isto é um verdadeiro mistério (M) " (E8 – 2).
	Expectativas	Apreensão com o Futuro	"Sabemos que ele não vai conseguir viver sozinho, vai ter sempre de ter um apoio […] enquanto cá estamos tudo bem, o problema é quando já cá não estivermos (M) " (E6 – 4).
	Estatuto Socioeconómico	Dificuldades Económicas	"Os valores das terapias no privado são altíssimos e as comparticipadas pelo estado são insuficientes […] o que torna tudo mais difícil (M) " (E1 – 2).
Eventos Significativos	Nascimento	Gravidez/Parto	"Durante a gravidez vive-se numa expetativa, mas quando eles nascem parece que superam aquela expetativa […] é uma emoção maravilhosa (M) " (E3 – 1).
		Amamentação	"Ele não queria mesmo mamar, foi uma luta desde o início […] Vim para casa e tive de falar para o SOS Amamentação porque foi um descalabre, não sabia o que fazer (M) " (E5 – 2).
	Confronto com um Comportamento Diferente	Falhas na Comunicação/Interação	"O meu filho não falava, embora emitisse uns sons (M) " (E5 – 3).<br>"Eu chegava do trabalho e ela ficava na mesma, era-lhe indiferente a nossa presença […] chegava a mudar de divisão quando estávamos presentes (P) " (E7 – 2).
		Estereotipias	"Começou a rodar objetos, a empilhá-los, a colocá-los em fila, não brincava ao faz de conta (M) " (E8 – 2).
		Preocupação dos Pais	"Eu estava muito preocupada porque me apercebia que havia qualquer coisa […] não sabia o quê, mas sentia, sabia que ele não estava bem (M) " (E1 – 3).
		Desvalorização das suas Opiniões	"Nós falámos das nossas desconfianças, inclusivamente de pensarmos em autismo, mas não nos valorizaram […] sentimo-nos muito sozinhos (M) " (E7 – 2).
	Encontro com a Realidade (Diagnóstico)	Choque	"É um balde de água fria" e nós não queríamos acreditar naquilo […] chorámos, […] parece que paralisámos (M) " (E8 – 4).
		Alívio	"Para nós foi um descanso porque a nossa maior angústia era não saber o que se estava a passar

			[…] foi a confirmação das nossas suspeitas e a partir daí já sabíamos o caminho a percorrer […] foi o começo do luto daquele filho perfeito que imaginámos ter (P) " (E6 – 5).
	Tomada de Consciência Face à Saúde	Escassa resposta dos profissionais	"É tudo muito burocrático […] nada facilitador no que se refere aos apoios, na informação, do que existe e como aceder (P) […] senti a falta de haver alguém (médico ou enfermeiro do centro de saúde) que me orientasse […] o Estado deveria acionar os apoios e deveriam encaminhar-nos e não sermos nós a termos de os procurar […] deveriam organizarem-se entre si (M) " (E7 – 2).
		Falhas de Comunicação entre Serviços	"Não há coordenação entre os serviços de saúde (médico e enfermeiro de família com os hospitais e serviços de apoio), não há encadeamento no acompanhamento […] isto não quer dizer que os profissionais sejam maus, o sistema é que não funciona (M) " (E10 – 1).
		Recursos Escassos	"As consultas no hospital para acompanhamento da [Estrela Polar] são anuais, imagine só uma vez por ano, como é possível? (M) " (E1 – 2).
	Tomada de Consciência Face à Educação	Pouco Envolvimento dos Profissionais	"Dentro da própria equipa não há comunicação e estratégias de ensino […] existem diretrizes educacionais na teoria, mas na prática é uma questão de sorte […] a escola não se esforça e não investe nestas crianças (M) […] O serviço não está a ser feito e estão-se a gastar recursos (P) " (E6 – 3).
		Reconhecimento da Vulnerabilidade dos Filhos	"Ouvimos falar sobre os abusos, os maus tratos […] muitas vezes a [EstrelaPolar] chega a casa com nódoas negras, o que é normal em qualquer criança, mas eu estou sempre com medo porque ele não fala, não consegue contar o que se passou e eu só tenho uma versão a dos adultos […] é angustiante (M) " (E1 – 4).
		Recursos Escassos	"Existem poucas professoras do ensino especial e terapeutas nas escolas para dar resposta às necessidades destas crianças (P) " (E2 – 3).
Vivência do Processo	Mudanças na Vida	Um novo olhar sobre a Deficiência<br>• Aceitação de um filho diferente	"O essencial foi perceber o que se passava com o [Cisne] […] foi passar por toda aquela fase de negação e progressivamente aceitar e integrar esse aspeto na minha vida e na vida familiar […] foi conseguir fazer opções, parar, pensar e decidir o que queria fazer da vida (M) " (E7 – 1).
		Adaptação a uma nova forma de vida<br>• Aprendizagens<br>• Envolvimento nos cuidados<br>• Mudanças nos hábitos/Alteração de rotinas	"A situação da nossa filha fez-nos mudar muita coisa […] veio trazer-nos uma nova forma de encarar a vida, mais ligeira, com outros valores (P) " (E7 – 2).<br><br>"Mudei o "chip" e pude combinar a vertente de cuidar com a vertente pedagógica […] reuni todo o material que tinha, aprendi como se trabalhava

	• Prevenção de situações embaraçosas	com estas crianças e avancei […] trabalhei muito com a [Cisne], fui mãe, professora, enfermeira, mas valeu a pena e ainda vale (M) ” (E7 – 1).<br><br>“Mudou muita coisa […] mudaram as nossas rotinas, tantas coisas que gostávamos de fazer (M) […] hoje somos um relógio e suíço (ri) […] temos de nos adaptar a ela (P) […] há muitas coisas que ficam adiadas (M) ” (E4 – 2).
	Alterações no relacionamento do casal<br>• Menor disponibilidade na relação<br>• Conflitos<br>• União/Amor	“O fato de nos apoiarmos mutuamente e de estarmos juntos é muito importante na relação (P) ” (E7 – 3).
	Modificações da vida social<br>• Isolamento<br>• Preocupação na manutenção da vida social	“A [Estrela Polar] tem algumas manifestações de contentamento que são reconhecidas como anti sociais […] os pais das outras crianças ficam a olhar para mim como se eu fosse uma mãe “desnaturada” […] é muito complicado gerir estas situações […] fica toda a gente a olhar para nós (constrangimento) (M) ” (E1 – 2).
	Modificações da vida profissional<br>• Opção de suspender a atividade profissional	”Foi também o momento da decisão de deixar de trabalhar porque era muito difícil conciliar o trabalho com as respostas que tinha de dar em casa […] naquele momento a prioridade era a [Cisne] e ela precisava de mim a tempo inteiro (M) ” (E7 – 3).
Características do Processo	Recursos/Mecanismos Internos<br>• Vivência do luto<br>• Espiritualidade	“Hoje em dia vejo a vida de outra maneira, vejo que há muito para ser vivido […] apoio-me numa coisa que é fundamental para mim que é o Amor […] o importante é ter forças para conseguir proporcionar felicidade aos meus filhos, e sentirmo-nos nós pais também felizes (M) ” (E4 – 2).<br><br>“A força surge no dia-a-dia […] é uma nova forma de se estar e ver o mundo […] (M) ” (E6 – 2).<br><br>”A força vem cá de dentro (M) ” (E8 – 1).
	Recursos/Mecanismos Externos<br>• Família/Amigos<br>• Associação<br>• Terapias/Serviços de Saúde e Educacionais<br>• Sociedade	“Estou sempre a dizer que nunca precisei tanto da minha mãe como agora, ela deita-me sempre a mão quando preciso (M) ” (E3 – 1)<br><br>“Deixámos de sair com frequência, mas mantemos contato com um casal amigo que é de toda a vida […] Temos mais intimidade e à vontade (M) ” (E8 – 2)

			"Graças a Deus que temos tido a Associação com pessoas que têm sido de uma importância extrema durante todo este percurso e têm-nos orientado de uma forma espetacular (M) " (E3 – 2).
Resultado do Processo	Autoconhecimento	Self/Competência Pessoal	"Temos aprendido tanto com toda esta situação (M) […] com o que temos aprendido adotámos a nossa maneira de lidar com ele e pensamos que esta é a melhor aproximação para com o [Belatrix], o que resulta melhor com ele […] Conseguimos avaliar a aprendizagem dele e a relação que tem connosco por isso vamos continuar nesta linha, embora estejamos sempre abertos a experimentar outras abordagens (P) " (E3 – 3).
	A vivência da experiência	Convivência	"Os livros falam do autismo, mas não ensinam a lidar com estas crianças […] só através da convivência diária […] da experiência […] é que compreendemos e aprendemos realmente […] é preciso estar-se lá e não há ninguém melhor que os pais (M) " (E1 – 1).
		Partilha	"A existência de grupos de apoio são muito importantes […] os pais têm tantas coisas em comum e nem sempre têm a oportunidade de se relacionarem uns com os outros e trocar ideias preciosas […] nem as nossas famílias nos percebem tão bem como os outros pais que vivem experiências semelhantes (P) " (E2 – 1).
	Domínio das novas competências	Satisfação e confiança no desempenho do papel parental	"Este esforço da nossa parte e o investimento feito tem tido recompensas e hoje olho para trás e apercebo-me que tomámos a opção correta e o importante que foi para o desenvolvimento da [Cisne] (M) […] todos os momentos do percurso foram importantes, foram uma aprendizagem e é por eles que continuamos e nos debatemos continuamente […] estamos confiantes e otimistas (P) " (E7 – 1).
		Eficácia	"Sabermos como antecipar alguns comportamentos, sabermos o que devemos fazer para eles estarem mais equilibrados, mais calmos é uma aprendizagem constante (M) " (E10 – 1).
	Redefinição do papel parental	Crescimento pessoal	"Acho que me tornei uma pessoa melhor, mais humana, mais calma, mais tranquila com mais paciência embora tenha muitas mais preocupações […] consigo gerir melhor as situações […] sinto-me hoje uma pessoa mais forte (M) " (E1 – 1).
		Melhoria na consecução do papel parental	"Hoje em dia vejo a vida de outra maneira, vejo que há muito mais para ser vivido […] é muito difícil ter um filho com necessidades especiais, mas hoje em dia já não dramatizo, vejo a vida de uma forma mais simples […] o importante é ter

		forças para conseguir dar felicidade aos meus filhos, e sentirmo-nos nós pais também felizes (M) " (E4 – 2).

Legend: PEA is the Portuguese translation of ASD. M – Mãe (Mother;) P – Pai (Father). The code after quotations refers to the interviews and codes of the analysis.
